# Building resilience against violent extremism digitally: trialing a new gender-based approach among gamers

**DOI:** 10.3389/fpsyg.2025.1537492

**Published:** 2025-07-28

**Authors:** Galen Lamphere-Englund, Mike Wilson, Jessica White, Claudia Wallner, Rachel Kowert, Nitchakarn Kaewbuadee, Petra Regeni, Alex Bradley Newhouse

**Affiliations:** ^1^Royal United Services Institute, London, United Kingdom; ^2^Love Frankie, Bangkok, Thailand; ^3^Department of Psychiatry, University of Cambridge, Cambridge, United Kingdom; ^4^Department of Political Science, University of Colorado Boulder, Boulder, CO, United States

**Keywords:** gaming, resilience, online communities, terrorism studies, violent extremism, gender

## Abstract

The rise of online gaming as a dominant social and entertainment space has increasingly attracted attention as a potential vector for radicalization to violent extremism. However, given the challenges of completely eradicating harmful content from these vast community spaces, we also need to focus on strengthening the resilience of individuals active there. To effectively build that resilience, we must first define and understand the current state of resilience among gamers, then identify the factors that contribute to it. This article seeks to do that by building upon the mostly offline-focused Building Resilience Against Violent Extremism framework to explore its applicability in the digital environment and specifically within the highly gendered parameters of gaming spaces and experiences. This article builds upon the wider data collection and findings of a project exploring socialization in gaming spaces with a nexus to radicalization through a gender lens.

## Introduction

The rise of online gaming as a dominant social and entertainment space has increasingly attracted attention as a potential vector for radicalization to violent extremism (VE). Gaming platforms, with their global reach and immersive experiences, are prime environments for fostering identity formation and community building—factors that can be exploited by extremist actors (Schlegel and Kowert, 2024; Lamphere-Englund and White, 2023). An essential part of countering and preventing the spread of VE in gaming and other online spaces is limiting the availability of extremist content and curbing the influence of extremist actors. Yet, given the challenges of completely eradicating harmful content from these vast community spaces, we also need to focus on strengthening the resilience of individuals active in online spaces against VE. To effectively build that resilience, we must first define and understand the current state of resilience among gamers, then identify the factors that contribute to it.

The article builds on data collected from the research project ‘Examining Socialization with a Nexus to Radicalization Across Gaming (−Adjacent) Platforms Through a Gender Lens,’ which was funded by Public Safety Canada, led by the Royal United Services Institute and implemented by a consortium of members of the Extremism and Gaming Research Network (EGRN). The article aims to bridge a gap in understanding the socialization processes in gaming spaces that contribute toward radicalization leading to VE. Specifically, we develop and test a modified version of the offline-centered Building Resilience Against Violent Extremism (BRAVE) framework (Grossman et al., 2017), revised for digital and gaming environments – calling it Building Resilience Against Violent Extremism Digitally (BRAVED). In doing so, we present a resilience model tailored specifically to the gaming space. Using data collected through an international survey of 2,244 gamers in Australia, Canada, France, Germany, Indonesia, the US, and the UK, this study presents a factor structure of digital resilience against VE, based on the core components of the BRAVE model. This framework allows for the categorization of survey participants into “Low,” “Mid,” and “High” resilience groups, providing insights into how various factors contribute to differing levels of resilience. The analysis across different demographic groups offers key insights into how resilience varies by country, age group, and gender, revealing valuable insights for targeted intervention activities to build resilience.

In doing so, this article contributes to the theoretical grounding of digital resilience frameworks. It provides practical insights for policymakers, game developers, and practitioners working to prevent and counter VE within gaming and gaming-adjacent platforms.

## Resilience against violent extremism in digital spaces

Resilience intersects with broad fields of study, as the concept originated in environmental ecosystem theory and expanded to fields as divergent as public health and terrorism studies. For this article, we define resilience as the ability of a group or individual to withstand exogenous shocks, particularly violent extremist ideologies and recruitment attempts, without employing violence (Grossman et al., 2017; Ungar, 2021). We also place the conceptualization of resilience at an individual level in negotiation with social ecosystems such as broader ethnic, religious, and gendered practices and norms, particularly social capital bonds such as those built through gaming communities (Bronfenbrenner and Morris, 2006).

In doing so, we align this study with the broader literature around resilience and “protective factors” (Rottweiler, 2022) against VE, which typically draw on institutional, social, and political processes across many systemic layers (Sieckelinck and Gielen, 2018). Meanwhile, in keeping with Berger (2018), we define extremism as the “belief that an in-group’s success or survival can never be separated from the need for hostile action against an out-group,” and VE as that which promotes ideological, political, or religious aims; advocates for or actively uses violence to realize those aims; and, tolerates, supports, or directly uses violence against civilians or critical civilian infrastructure.

This study draws on the BRAVE framework and survey tool (Grossman et al., 2022, 2017). In keeping with the definition of resilience above, BRAVE looks at not merely the absence of risk factors or specific correlations to support for violent extremism, but looks at a composite and positive set of protective factors that empower individuals and communities (Lamphere-Englund et al., 2022). BRAVE is designed to be localized and adapted to each cultural context in which it is deployed and has been used in over 15 countries to date, including studies in Indonesia and Kyrgyzstan (for example, Jailobaeva, 2020; Lamphere-Englund et al., 2022). This is, to our knowledge, the first attempt at adapting BRAVE to a specific set of digital communities.

The framework is built on interrelated factors that act as protective factors against VE: cultural identity and connectedness, bridging capital, linking capital, and violence-related beliefs and behaviors. These factors collectively capture a range of identity complexities and social capital measures (Putnam, 2000, 2001) that underpin an individual’s ability to resist extremist narratives. BRAVE measures protective positive attributes conceptualized to guard against VE. However, less of these protective factors at an individual level should not be viewed tautologically as presenting a higher likelihood of committing targeted violence or terrorist acts. It is not a behavioral threat assessment tool at a granular level; but rather a macro-level view of what can strengthen communities.

On this foundation, we provide a novel, experimental adaptation of BRAVE by adapting it for digital and gaming environments: BRAVED. The next sections describe, in brief, the theoretical underpinnings of core factors of the model, while the full survey questions can be found in Annex 1.

## Theoretical framework components

### Cultural identity and connectedness

Cultural identity and connectedness in BRAVE encompass how individuals maintain and engage with their cultural background(s), including their sense of belonging to cultural groups and confidence in navigating diverse cultural environments (Grossman et al., 2017). This factor is critical in forming a positive sense of self, which can act as a buffer against extremist narratives that often exploit feelings of identity-based grievance or exclusion (Phelan, 2023). Within digital spaces, especially gaming environments, cultural identity plays a pivotal role in determining how individuals interact with others and perceive the inclusivity of online communities (Wallner et al., 2023). The project highlighted the importance of considering the gendered and intersectional nature of identity and how socio-culturally developed identity factors impact gamers’ individual and community identity formations and behaviors (White et al., 2024).

In this study, we adapted BRAVE’s focus on cultural identity to digital contexts by asking respondents how identities such as religion, ethnicity, gender expression, and sexual orientation play into online interactions and how strongly they feel grounded in those identities. We also ask how strongly individuals identify with being a gamer and how that identity interplays with the more traditional aspects above. Considering these dimensions, our approach acknowledges the multiple and intersecting identities that shape players’ experiences in a gaming environment. Respectively, examining the role of religion in shaping behavior and experiences within these spaces offers insight into how religious identity – along with knowledge, which we did not determine in this study – can influence resistance to extremist narratives (Fair et al., 2017; Fair and Patel, 2022). Ethnicity was included as a critical part of identity formation, as well as a group-identity-building characteristic used both for beneficent and malicious ends: for example, support for white supremacy in gaming communities (Kowert et al., 2022). Prior research shows that women and marginalized gender and sexual identities often face heightened harassment online, affecting their sense of belonging and resilience pathways (Fox and Tang, 2017). Lastly, we included a new variable to the identity matrix: that of being a gamer. A distinct “gamer” identity is a powerful marker within digital spaces. Prior research – including by members of our research team – has shown how individuals express and fuse complex layers of their personhood into a singular gamer identity, which in turn appears to reduce resilience against extremism (Gómez and Vázquez, 2015; Kowert et al., 2022).

We also sought to improve the standard BRAVE framework by grafting the survey into a broader Gender-based Analysis Plus (GBA+) framework, which we used to examine all elements of data collection and analysis in the other forthcoming project outputs (White et al., 2024; Schlegel and Kowert, 2024; Wallner et al., 2023). The framework was used at every stage of the project to ensure that analysis of gender identity and the full spectrum of intersecting identity factors— including race, class, age, ability, sexuality and geography—were not treated as an after-thought but as core analytical lenses. GBA + ‘s “Plus” reminds us that radicalization pathways are shaped by overlapping systems of privilege and marginalization, not by gender alone (Government of Canada’s Approach on Gender-Based Analysis Plus,” Government of Canada, Women and Gender Equality Canada (WAGE), 2024). Guided by this principle, we treated violent misogyny, homophobia, and other forms of targeted hate as mutually reinforcing risks that can weaken individual and community resilience. Survey items therefore both captured respondents’ experiences of inclusion and harassment, while also exploring how in-group bonding and out-group “othering” differ across platforms and player demographics.

Applying the framework meant designing a deliberately global, cross-cultural sample within budgetary limitations. We chose to look at Canada, the US, the UK, France, Germany, Australia and Indonesia to surface how the same game mechanics and social features can have divergent equity impacts in distinct regulatory and cultural contexts. Phase One of the broader project generated the evidence base; Phase Two translated those insights into this article, along with a practitioner toolkit and policy briefs. Throughout, we asked not only *who* is most exposed to extremist narratives in gaming, but also *why* intersectional identity profiles may face distinct challenges and benefit from tailored safeguards.

### Bridging capital

Bridging capital represents the social interactions and relationships across different groups of people. It reflects the inclusiveness and openness of an individual’s social network (Putnam, 2000). The ability to actively engage with diverse others can foster tolerance and empathy, which are, in turn, essential components of resilience against extremist narratives that seek to polarize communities (Miller-Idriss, 2020). In gaming contexts, we translated the concept of bridging capital into the comfort levels and preferences gamers have when interacting with others from various identities. We note that the ability to form loose and close ties within diverse gaming communities has been found to contributes significantly to building resilience (Kowert et al., 2022).

### Linking capital

Linking capital refers to how individuals relate to institutions and authorities, such as their trust in law enforcement or government agencies (Grossman et al., 2022). This factor indicates individuals’ comfort and confidence when engaging with power structures. While not directly measured in our adaptation to digital spaces, linking capital remains relevant, as the gaming community often interacts with platform moderators, law enforcement, and policy frameworks that govern online behavior (Lamphere-Englund and Hartgers, 2024). Understanding this interaction is vital to addressing toxic behavior and harassment within online gaming spaces (Klein and Nocera, 2024; Anti-Defamation League, 2024). In our survey, we looked at reporting knowledge and feelings of being heard when reporting in gaming spaces, but after review decided these did not directly map onto linking capital and chose to report on those in separate reports (forthcoming in 2025).

#### Confronting hate

Confronting hate, or the willingness to stand up for others being harassed or harmed, is a proxy for and corollary to bridging capital and, to an extent, linking capital. We assess this as a separate factor in the analysis, though the theoretical underpinnings are closely associated with social capital theory. For our online adaptation, we asked about players’ willingness and actions to stand up against harassment and extremist content in gaming spaces. The presence of such behaviors signals a level of social cohesion and collective resilience that can counter divisive extremist narratives (Anti-Defamation League, 2024). Research has indicated that when individuals actively confront hate online, it can create a more inclusive and resilient community (Jakubowicz et al., 2017; Wachs et al., 2024; Wallner et al., 2023).

### Violence-related beliefs and behaviors

BRAVE also examines attitudes toward violence, capturing beliefs that justify violence and behaviors that challenge violent actions (Grossman et al., 2022). This factor reveals the cognitive and behavioral dimensions of resilience by assessing how individuals confront or resist narratives promoting VE. In gaming spaces, these attitudes often manifest as reactions to toxicity, harassment, and extremist content, where players may either condone or confront such behaviors (Schlegel and Kowert, 2024). We looked at two proxies for these attitudes:

1. Intolerance and Intolerant Beliefs: In gaming environments, we used this as a proxy for “violence-related beliefs” in the original BRAVE tool. This category captures respondents’ attitudes toward toxicity and extremist content in gaming spaces. By exploring these dynamics, we aim to understand how normalizing toxic behaviors can affect gamers’ perceptions and susceptibility to extremist narratives (Schlegel and Kowert, 2024). The online gaming ecosystem has been found to both propagate and challenge these intolerant beliefs, depending on the nature of player interactions (Lamphere-Englund and White, 2023).
2. Hate and Harassment-Related Behaviors: We use these as an online proxy for violence-related behaviors asked for in BRAVE. Digital gaming environments are known to host a spectrum of abusive behaviors, including identity-based harassment (Fox and Tang, 2017). Understanding to what extent respondents engage in these behaviors within the context of gaming spaces allows us to draw then correlations based on a willingness to engage in identity-based harassment that might shape individuals’ resilience to extremist ideologies. This focus aligns with research showing that exposure to and participation in toxic online behaviors can impact one’s likelihood of adopting extremist views (Phelan, 2023).

## Materials and methods: developing and testing BRAVED

### Data collection

#### Survey design

To assess exposure, vulnerabilities, and resilience to VE in gaming and gaming-adjacent platforms, we designed a comprehensive survey instrument deployed across seven case study countries: Australia (AU), Canada (CA), Germany (DE), France (FR), United Kingdom (GB), Indonesia (ID), and the United States (US). The survey’s primary goal was to address the research questions outlined in the introduction, particularly evaluating how gamers’ resilience to VE is influenced by factors such as exposure to extremist content, gender, cultural differences, and gaming behavior.

The 20-question survey included questions we used to develop our compound BRAVED measure. Additional questions were added on exposure to violent extremist content, normalization of toxicity and extremism in gaming spaces, games played, adjacent platform use, and experiences with harassment but were not used in this analysis [those findings can be found in White et al. (2024)]. We also explored the role of gender and cultural variances across geographies in shaping the socialization process within transnational gaming spaces.

By drawing on the principles of the BRAVE framework, as outlined above, we created the BRAVED compound measure of resilience. The exact questions and factors used are elaborated below.

The survey design involved multiple stages of validation. The survey instrument was developed in consultation with the research team and EGRN partners, with extensive internal reviews to ensure relevance across different contexts. After the initial design, the survey was translated into the languages of the case study countries and underwent a thorough review for accuracy in terminology. A pre-launch phase involved testing with partner organizations to refine the appropriateness of language, length, and sensitivity of topics. The research adhered to robust ethical standards and institutional ethics approvals governing sensitive data collection and reporting throughout the implementation.

#### Survey implementation

The survey was conducted through mobile online polling via Pollfish, a private polling provider. Pollfish uses a random sampling of pre-screened mobile phone users in a more extensive survey panel maintained by the company. Respondents were invited through a double opt-in process to maintain rigor in respondent eligibility. Each country was assigned a soft quota targeting *n* = 300 respondents (split evenly by gender, with an age distribution of 18–28 and 29–44). The survey ran from August 4-28, 2023, yielding a total sample size of *n* = 2,244 across all seven countries (Table 1).

**Table 1:** Survey sample.

Country	Sample (*n*)	Sample (%)
AU	320	14.3
CA	336	15.0
DE	300	13.4
FR	300	13.4
GB	352	15.7
ID	300	13.4
US	336	15.0
TOTAL	**2,244**	**100**

### Informed consent and ethics

The research plan, survey instrument, and data-handling rules received full approval from two Institutional Review Boards involved in the full study of which this article is one output. As noted, the project was funded by Public Safety Canada, led by the Royal United Services Institute (RUSI) and implemented by a consortium of members of the Extremism and Gaming Research Network. All fieldwork was cleared by the Royal United Services Institute Ethics Committee and the University of Southampton’s ERGO panel (ERGO/92202). All work complied with relevant counterterrorism regulation and were found to fully address ethical issues. The survey ran through a commercial service provider – Pollfish – and it’s random-device-engagement network, which sends ads to adults inside thousands of mobile apps and invites them—once only—to participate. Before answering a single question, each prospective respondent sees a standalone informed consent screen that (i) explains the study’s purpose and expected length, (ii) stresses that participation is voluntary and can be terminated at any time, (iii) outlines how responses are anonymized, and (iv) offers a small in-app incentive. Continuing requires an explicit “I agree” tap; otherwise the invitation closes with no data captured. Pollfish blocks bots and duplicates, disqualifies anyone who reports being under 18 or who fails age-screeners, and operates under GDPR, CCPA and ESOMAR/MRS rules, while sharing no personally-identifiable information with researchers.

Downloaded survey data contained no names, emails, IP addresses or advertising IDs. They were encrypted immediately and stored on password-protected institutional servers; working files resided only on VPN-protected, dedicated laptops. All public-facing analyses aggregate the data and redact free-text that could re-identify individuals. Researcher welfare was also ensured by ethics protocols: exposure to violent or hateful responses was time-limited, tasks were rotated, and staff had access to mental-health support. These combined safeguards ensure the project met international standards for informed consent, privacy, data security and duty of care.

### Limitations

First, the generalizability of our survey sample is limited due to our reliance on an opt-in, mobile-based survey of 2,244 gamers recruited through Pollfish across seven countries and two age brackets (18–28; 29–44). Although quotas were set for gender and age, the sample is not probability-based: respondents self-select into the panel and may differ systematically from gamers who do not engage with in-app polling.

Second, as all measures were self-reported, social desirability bias due to the sensitive nature of asking after hate and harassment behaviors be at play. In the full study (White et al., 2024), we collected and analyzed primary data from online sources to mitigate that effect, but this article only relies on the survey data for analysis. Even with anonymity, recall errors, social-desirability bias and differential willingness to disclose controversial behaviors (e.g., harassment) means that respondents may down-play their own misconduct. In short, this means the survey may understates the true prevalence of hate and harassment reported elsewhere in gaming research. Future studies should test indirect questioning, endorsement experiments, or list-experiment techniques to gage these behaviors more accurately. We chose to focus on the BRAVED measure and not draw on external behavioral data for this limited article.

Third, the survey offers only a snapshot in time captured between 4 and 28 August 2023. Gaming cultures, platform policies, and extremist tactics evolve rapidly. Resilience levels and exposure patterns may have shifted since data collection. Longitudinal or repeated-cross-section designs would be required to track trends over time and to assess causality in the exploratory relationships identified here.

Fourth, while the use of a mobile polling method ensured geographic reach, it provides no visibility into private or encrypted game chats where socialization linked to extremism and mobilization often occurs. Our findings here relate to gamers’ perceptions and self-reported experiences rather than to the full ecology of in-game interactions or platform-level moderation data. For exploration of those wider dynamics, please see the longer analysis from this study (White et al., 2024).

Finally, the findings below, and especially those drawn between countries should be viewed as exploratory in nature. The study was powered primarily to validate the BRAVED factor structure; the per-country cell sizes (*n* = ~300) limit statistical power for fine-grained subgroup tests, and we did not control for all contextual variables (such as national gaming-market composition, regulatory environments, and so on). Consequently, observed cross-national differences in resilience or factor loadings like the higher “High-resilience” share in the United States versus France and Germany should be interpreted as indicative patterns that warrant deeper, country-specific investigation rather than definitive rankings.

### Exploratory factor analysis (EFA) and reliability test results

We conducted an Exploratory Factor Analysis (EFA) following data quality checks. The primary purpose of this analysis was to identify latent factors that could explain the patterns of responses across the multiple survey items related to exposure to hate, harassment, extremist content, and other variables associated with gamer identity and socialization. This approach allowed us to substantiate our theoretically grounded framework for resilience, which was used to develop a compound resilience indicator.

Before running the analysis, we verified the suitability of the data for factor analysis by examining measures of sampling adequacy. Using a Kaiser-Meyer-Olkin (KMO) test yielded values above 0.80 for most of our model variables, indicating an appropriate level of sampling adequacy. Bartlett’s test of sphericity was significant (*p* < 0.001), confirming that the data was suitable for factor analysis. Principal axis factoring (PAF) was used to extract factors. Eigenvalues and scree plots were employed to determine the number of factors to retain from our 20 questions (and sub-questions). Based on the results, we grouped our results into seven factors. We applied an oblique rotation (Promax) to improve interpretability. Given that the factors are likely correlated (e.g., exposure to hate and harassment behaviors likely correlates with other forms of intolerance), the oblique rotation was deemed more appropriate than orthogonal methods. Each survey item was loaded onto its respective factor, with a threshold set at 0.40 for inclusion.

The EFA results confirmed the presence of distinct, interpretable factors that aligned with our theoretical framework – a seven-factor model that explained most of the variability in our data. However, some items did not load as expected and required further refinement or exclusion from subsequent confirmatory analyses. For example, items related to bullying behaviors (Factor 2) loaded strongly together, while items concerning tolerance toward others in gaming spaces (Factor 3) showed a similar internal consistency.

To ensure the internal consistency of the identified factors, we conducted Cronbach’s alpha reliability tests for each of the seven factors. Cronbach’s alpha measures internal consistency, indicating how closely related a set of items is as a group. Factors with a Cronbach’s Alpha above 0.70 were deemed acceptable, though we considered factors below this threshold for inclusion based on theoretical importance. The final factors were:

*Factor 1: Hate and Harassment-Related Beliefs,* which included items related to toxicity, extremism, and difficulties in trusting others from diverse backgrounds online. The items measuring attitudes toward toxicity and extremism showed high internal consistency: Cronbach’s alpha was 0.647. Questions included:

- Q17_1: “I think toxicity in the gaming space is…[Unacceptable, Normalized, Culturally Acceptable, Justified]”
- Q17_2: “I think extremism in the gaming space is…[Unacceptable, Normalized, Culturally Acceptable, Justified]”

*Factor 2: Hate and Harassment-Related Behaviors,* reflecting individual self-reports of harassment and bullying based on protected identity markers such as religion, race, gender identity, and sexual orientation. This factor displayed Cronbach’s alpha score of 0.769, indicating internal solid reliability across questions about bullying behaviors. This was a multiple-selection question:

- Q13: “I have bullied or harassed people based on their…” [Age, Class, Ethnicity/race, Nationality, Religion, Gender identity/expression, Sexual orientation, or All of the above.]

*Factor 3: Bridging Capital,* which looked at the reported levels of comfort in interacting with people of diverse identities, both in broader gaming spaces (Loose Ties - 3a) and in forming meaningful, strong relationships (Close Ties – 3b). This was split into two factors following EFA. The internal consistency of 3a (Loose Ties) was high at 0.857, with Factor 3b (Close Ties) sub-factor also internally consistent at 0.695. Questions in each included:

- *Loose Ties (3a),* consisting of Q8: “I feel comfortable playing with people of different identities, including different…” [Same identity options as above, and “only people that share my identity].
- *Close Ties (3b),* consisting of two questions: Q10_2, “I regularly engage in conversations online with people with different identities or backgrounds,” and Q10_3, “I have strong and meaningful friendships online with friends from different identities than mine”

*Factor 4: Confronting Hate,* including items about recognizing extremist content, reporting harmful behavior, and standing up for others being harassed. This factor had acceptable internal reliability (Cronbach’s alpha = 0.680), confirming that knowledge of and action against hate in gaming spaces was consistently measured. Questions and responses in this factor included:

- Q16_2: “I can tell the difference between commonplace toxicity and extremist content online.”
- Q16_3: “I can tell the difference between online sexism and misogyny.”
- Q20_2: “I have reported harmful content in the last year.”
- Q22_1: “I have stood up for someone else being harassed online from my own community.”
- Q22_2: “I have stood up for someone else being harassed online from a different community.”

*Factor 5: Cultural Identity and Connectedness – Religion and Ethnicity,* which looked at how those identity aspects shaped respondents’ behavior and sense of belonging in gaming spaces. The internal consistency for this factor was slightly lower than expected (Cronbach’s alpha = 0.619), likely due to the heterogeneity of the items included. Further revisions were flagged, and we tested models with separate Religious and Ethnic identity factors, which improved the model fit at the expense of internal reliability. As such, we ultimately chose to keep this as one factor. Questions and responses that we built into this factor included:

- Q6_4: “The way I am living my life is guided by the traditions, beliefs, habits, and norms of my culture/ethnic group.”
- Q7_3: “My gamer identity represents the following: Ethnicity/race.”
- Q9_3: “I have actively sought/found communities based on similarity to my Ethnicity/race.”
- Q6_5: “Religion is important for me and guides me on how to behave in society.”
- Q7_5: “My gamer identity represents the following: Religion.”
- Q9_5: “I have actively sought/found communities based on similarity to my Religion.”

*Factor 6: Cultural Identity and Connectedness—Gender and Sexual Orientation,* revealing how individuals’ community engagement reflected these aspects of their identities. This factor showed lower internal reliability (Cronbach’s alpha = 0.591), but as a critical factor for the gender component of the study, we elected to keep it in the model. Notably, the larger study framework specifically focuses on gendered experiences of gaming identity, and qualitative research from other studies have noted the striking differences in experience of hate and harassment faced by different genders in gaming. As such, the research team chose to retain Factor 6 in the model. Questions and responses included:

- Q7_6: “My gamer identity represents the following: Gender identity/expression.”
- Q9_6: “I have actively sought/found communities based on similarity to my Gender identity/expression.”
- Q7_7: “My gamer identity represents the following: Sexual orientation.”
- Q9_7: “I have actively sought/found communities based on similarity to my Sexual orientation.”

*Factor 7: Cultural Identity and Connectedness—Gamer Identity,* looking at how representative one’s gamer identity was of other identity factors, including age, class, ethnicity, nationality, religion, gender, and sexual orientation. The reliability for this factor was weaker than the others (Cronbach’s alpha = 0.373) and would be considered unacceptably in terms of overall internal reliability. Given its importance to the study, we retained it for confirmatory factor analysis, but further revisions were considered. We ran confirmatory factor analyses with and without the gamer identity factor included, which did not distort the overall findings presented below. As gamer identity fusion has been posited as a predictor of support for extremist beliefs and dark triad behaviors, we also felt it was justifiable to retain the factor in final analysis (Kowert et al., 2022).

- Q7: “My gamer identity represents the following…[Age, Class, Ethnicity/race, Nationality, Religion, Gender identity/expression, Sexual orientation, or All of the above]”

The reliability tests affirmed the validity of most factors, though some (e.g., Factors 6 and 7) required additional scrutiny. Merging all identity factors into one factor significantly improved internal reliability (Cronbach Alpha score of 0.79), but created an unreliable overall factor model. Ultimately, we elected to use the above seven factors and one sub-factor (3b) above in the final BRAVED model.

## Results and analysis

### Confirmatory factor analysis (CFA)

After ensuring internal construct validity, we conducted Confirmatory Factor Analysis (CFA) in R to test our hypotheses: do the composite factors of create a valid model and, if so, which specific factors have the greatest effect on the overall composite measure? CFA is a statistical technique that allows researchers to examine the relationships between observed variables and their underlying constructs (the questions). CFA was carried out using the entire dataset collected from seven countries via using the ‘lavaan’ package in R.

In CFA parlance, we hypothesized that resilience against VE is a second-order latent variable influenced by the first-order latent constructs, which are represented by the seven factors and one sub-factor. We used standardized factor scores to adapt for the variation in how many questions were included in each factor. Also, we added covariances for factors we expected would correlate, such as “Bridging Capital” with “Confronting Hate,” based on theoretical expectations from the literature on resilience.

Our use of CFA allowed us to assign relative resilience scores and categorize participants into “Low,” “Mid,” and “High” resilience groups based on the mean and standard deviation of their “Resilience” scores. Overall, the CFA fit indices are acceptable, suggesting that our model captures the underlying factor structure and aligns with our theoretical framework.

In terms of measures of fit: Comparative Fit Index (CFI) was 0.905, indicating a good fit, as values closer to 1 are ideal. A CFI value above 0.90 is generally considered acceptable. The Tucker-Lewis Index (TLI) was 0.896. Which is slightly below the commonly accepted threshold of 0.90 for a good fit, indicating some room for model improvement but close enough to a good value to provide merit. Meanwhile, RMSEA was 0.039, with a 90% confidence interval of 0.038 to 0.041. An RMSEA value below 0.05 suggests a close fit, supporting the model’s adequacy. Lastly, the Standardized Root Mean Square Residual (SRMR) was a very acceptable 0.046. SRMR values less than 0.08 are considered robust.

### Primary drivers of resilience

**Figure 1:**
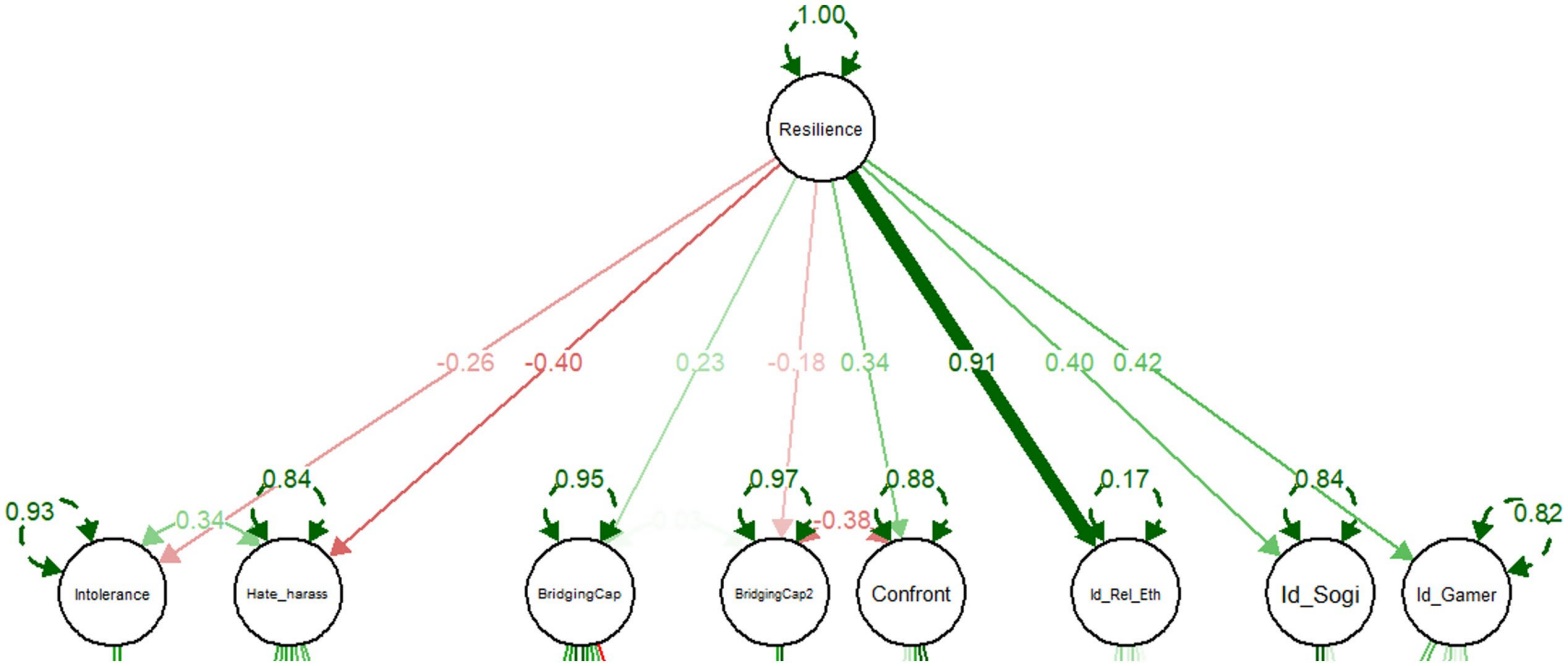
CFA factor loadings.

Table 2 below and Figure 1 above outline the second-order factor loadings in the Confirmatory Factor Analysis (CFA), detailing the relationship between factors (Intolerance, Hate, Harassment, Bridging Capital, etc.) and the overarching composite resilience against violent extremism construct. As a reminder, that composite measure is posited to include all the below factors as protective (positively coded and contributing to resilience) in nature exception of with hate-and-harassment related beliefs and behaviors (inversely, negatively coded and reducing resilience). These factor loadings help us understand how each contributes to our overall composite model of resilience to VE. The below analysis is presented in order of each factor’s strength of contribution grouped by protective (positive) factors and negative factors that reduced overall resilience.

**Table 2:** Table of factor loadings and reliability figures.

Factor	Impact on composite resilience	SE	*Z*-score	*p*-value	CI lower	CI upper	Std. Dev.
Hate and harassment-related beliefs	−0.2745	0.0361	−7.6076	0.0000	−0.3452	−0.2037	−0.2647
Hate and harassment behaviors	−0.4385	0.0370	−11.859	0.0000	−0.5110	−0.3661	−0.4016
Bridging capital (Loose Ties)	0.2342	0.0298	7.8626	0.0000	0.1758	0.2926	0.2281
Bridging capital (Tight Ties)	−0.1841	0.0322	−5.7242	0.0000	−0.2472	−0.1211	−0.1811
Confronting hate	0.3628	0.0325	11.1574	0.0000	0.2990	0.4265	0.3410
Cultural identity: religion and ethnicity	2.1791	0.5305	4.1079	0.0000	1.1394	3.2188	0.9089
Cultural identity: sexual orientation and gender expression	0.4347	0.1304	3.3333	0.0009	0.1791	0.6903	0.3986
Cultural identity and connectedness — gamer identity	0.4667	0.0896	5.2110	0.0000	0.2911	0.6422	0.4229

Red and orange: Negative impact on resilience. Yellow and Green: Positive impact on resilience.

### Protective factors

Respondents who felt their lives – both in games and outside of them – were rooted in cultural and religious identities tended to have higher overall resilient scores than others. The compound religious and cultural identity factor had the greatest overall positive impact on resilience (+2.18) with high statistical significance. Based on our scorings, that meant that individuals with greater religious and ethnic identity and connectedness were less likely to exhibit hate and harassment-related beliefs and engage in targeted harassment based on protected characteristics. At the same time, they were more likely to have strong social capital ties (Bridging Capital) and to stand up to defend others online (Confronting Hate). We found that religious and cultural identities were intensely correlated, grouping them into a single factor for this analysis.

Other identity-based factors were the following most substantial protective factors in this study. The more respondents felt their gamer identity represented other identity characteristics, the more resilient they became across all other factors (+0.47). We asked how reflective their gamer identity was of their age, class, ethnicity/race, nationality, religion, gender identity/expression, and sexual orientation. A complex and multifaceted gamer identity, encompassing different aspects such as age, gender, ethnicity, and nationality, enhances overall resilience. We interpret this as the opposite effect of gamer identity fusion – in which a flattening of gamer identity occurs as it becomes the dominant identity factor and other layers matter less. Previous work by Kowert et al. (2022) has shown that such a fused identity correlates with increased support for VE (white supremacy, in particular). This builds on a wider evidence base inside which identity fusion is typically associated with susceptibility to violent extremist ideologies and organizational support (Gómez et al., 2011; Swann et al., 2009).

Finding rootedness and communities related to one’s gender expression and sexual orientation was also correlated with increased resilience in the model (+0.44). This was less significant (*z*-score = 3.33). However, we believe it is still robust enough to merit reporting on. Our question set looked at to what extent respondents’ gamer identity was reflected in their sexual orientation and gender expression and whether they actively sought out and found communities based on those identities (Connectedness). As this was also correlated with increased resilience across all factors, it appears that recognizing and supporting these identity layers matter for building resilient, supportive communities against VE.

We examined confronting hate in gaming communities both in terms of knowledge (knowing the difference between toxicity and VE, as well as between run-of-the-mill sexism and misogyny), as well as in terms of action (reporting harmful content and standing up for others being harassed). Unsurprisingly, a higher propensity to confront hate increased overall resilience, though to a lesser extent than the identity factors (+0.36). Regardless, this finding supports a commonsense correlation that actively standing up against harassment and extremist content in gaming spaces is crucial for enhancing digital resilience.

Lastly, in terms of protective factors, we found that loose connection forms of bridging capital also contributed to overall resilience (+0.23). We conceptualized loose social capital ties, conned by Granovetter (1973), as manifested in gaming settings by individuals comfortable playing with people of different identities (age, class, ethnicity/race, nationality, religion, gender identity/expression, and sexual orientation, or all of the above). This suggests that interactions with diverse individuals in gaming environments can build tolerance and empathy, similar to how diverse neighborhoods in urban settings tend to reduce racism and prejudice gradually (Tables 3–5).

**Table 3:** Resilience scores by country.

Country	Segment	Mean resilience score	Percentage of segment in country	Country level resilience
**AU**	Low	−0.87	8.75	0.07
**AU**	Mid	−0.20	73.44	
**AU**	High	1.67	17.81	
**CA**	Low	−0.86	6.25	−0.04
**CA**	Mid	−0.23	79.17	
**CA**	High	1.35	14.58	
**DE**	Low	−0.88	8.33	−0.20
**DE**	Mid	−0.33	81.67	
**DE**	High	1.46	10.00	
**FR**	Low	−0.87	14.67	−0.26
**FR**	Mid	−0.35	77.33	
**FR**	High	1.81	8.00	
**GB**	Low	−0.88	13.35	−0.07
**GB**	Mid	−0.28	71.88	
**GB**	High	1.69	14.77	
**ID**	Mid	−0.04	86.33	0.17
**ID**	High	1.50	13.67	
**US**	Low	−0.85	2.68	0.31
**US**	Mid	−0.12	71.43	
**US**	High	1.60	25.89	

**Table 4:** Resilience segments by age.

Age group	Resilience group	Mean resilience	Percentage of group	Resilience of age group
18–28	Low	−0.86	5.31	0.04
18–28	Mid	−0.18	80.15	
18–28	High	1.58	14.55	
29–44	Low	−0.88	10.08	−0.04
29–44	Mid	−0.27	74.20	
29–44	High	1.59	15.73	

**Table 5:** Resilience by gender.

Gender	Group	Mean resilience	Percentage	Resilience by gender
Male	Low	−0.87	6.45	0.03
Male	Mid	−0.20	77.89	
Male	High	1.57	15.67	
Female	Low	−0.88	9.05	−0.03
Female	Mid	−0.25	76.31	
Female	High	1.59	14.64	

### Negative factors

On the other hand, and somewhat surprisingly, we found that higher levels of close-knit forms of bridging capital *reduce* overall resilience (−0.18). While the overall impact was less than that of other actors, it was statistically significant. We asked whether people had regular conversations online with people from different identities and backgrounds and if they had strong and meaningful friendships online with those types of people. Potentially, this could indicate that social contact theory has limitations in online settings – namely when those points of contact go beyond gaming and extend into regular conversations. However, this effect was highly dichotomous across countries in the sample. In Canada, close ties were associated with a + 0.43 impact *positive* increase in resilience. All other countries saw a negative impact, but the degree varied from nearly inconsequential in the US (−0.005) to profoundly negative in Indonesia (−0.47) and the UK (−0.39). Perhaps, in some settings, deeper engagement breeds hostility. In contrast, in others, it encourages the creation of pro-social bonds. Further research on this point in virtual settings is needed.

Lastly, and rather uncontroversially, we found that holding both hate and harassment-related beliefs (−0.27) and behaviors (−0.44) in gaming settings were strongly and negatively associated with a decline in overall resilience. Specifically, we coded finding extremism and toxicity in gaming settings as culturally acceptable or justified as a hateful belief. Meanwhile, we coded self-reported bullying or harassing people based on a protected identity characteristic as hateful behavior. This aligns with existing research suggesting that intolerance, including toxic attitudes, increases susceptibility to extremist narratives. This finding underscores the importance of addressing and mitigating hate and harassment in digital spaces to foster a more resilient gaming community. Participation in or support of such targeted hate appears to weaken a host of other protective factors.

## Discussion

### Resilience by country, age, and gender

We also examined variations in the contributions of each factor across particular demographic groups in our study, namely across country, age group, and gender.

Importantly, we must note that these cross-country findings are purely exploratory in nature at this stage and do not account for a wide range of exogenous factors potentially impacting the scores in each country.

Across most countries, the majority of participants fall into the “Mid” resilience group, with percentages generally ranging from 71 to 86%. This pattern indicates a relatively moderate level of resilience across these countries. The results also provide a general resilience index for each country. Positive values (e.g., Australia, Indonesia, and the United States) indicate higher resilience, while negative values (e.g., Canada, Germany, France, and Great Britain) suggest a comparatively lower resilience among gamers that country.

The US and Indonesia are outliers in this study, exhibiting considerably higher resilience scores overall. In particular, both show notably higher percentages of highly resilient respondents (13.67% for ID and 25.89% for the US) than other countries. We also note that both of these countries were also those with the highest levels of exposure to violent extremist content and fundraising requests among our sample (a variable we discuss in our forthcoming reports on this dataset), suggesting that the protective effect of resilience in both could be even more substantial. We did not, however, see a statistically correlated relationship between exposure to violent extremist content and resilience, belying a causal relationship. Country-specific nuances in the two most resilience countries in our sample included the following:

*In the US*, possessing hateful beliefs (viewing toxicity and extremism as accepted or justified features of gaming communities) was nearly four times as negatively impactful on overall resilience than elsewhere (−0.97 in the US versus −0.28 across all countries). Meanwhile, having bridging capital was far more essential to increase resilience in the US than elsewhere (+0.41 in the US for loose ties versus +0.23 elsewhere, and −0.005 for close ties versus −0.18 elsewhere). This suggests, perhaps, that when American gamers find a connection with others, it is potent in inoculating them against extremist ideological entreaties. But those who have hateful beliefs in the US are far more likely to see a precipitous loss in overall resilience: the highs are high, and the lows are low in America.

*In Indonesia*, meanwhile, possessing hateful beliefs (−0.11 vs. -0.27 across the sample) and engaging in hateful harassment (−0.20 vs. -0.44 across the sample) were both less impactful on overall resilience. Effects of other factors were also more muted, with religious and ethnic identity and connectedness contributing just +0.40 instead of +2.18 across all countries. Yet Indonesians were extremely unlikely to have low resilience scores overall. The bottom of the distribution curve effectively did not exist for Indonesia, with respondents falling into our mid- (86% of respondents) and high-resilience brackets (14% of respondents). Thus, the average resilience of Indonesian gamers is all the more striking.

The country-specific breakdown of resilience scores reveals variations that could be tied to cultural, societal, and digital ecosystem differences in each location. For instance, the higher “High” resilience percentage in the US might reflect the influence of community support systems or digital literacy efforts within its gaming spaces. On the other hand, countries like France and Germany show a lower overall resilience index, pointing to potential areas where interventions might focus on enhancing digital resilience, particularly among youth. Other country-level differences may be explored in another analysis of this dataset.

### Resilience by age

The “Mid” resilience group dominates both age categories, with a slightly higher percentage in the 18–28 group (80.15%) compared to the 29–44 group (74.20%). The percentage of individuals in the “High” resilience group is somewhat more numerous in the older 29–44 age group (15.73%) than in the 18–28 group (14.55%). This indicates that a slightly higher proportion of older gamers may possess stronger resilience against extremist narratives in digital spaces. However, taken on average, the 18–28 age group (0.04) has a marginal but statistically significantly higher overall resilience than the older group (−0.04).

When we examine specific factor differences between our younger and older cohort, we find that:

- Holding hate and harassment-related beliefs and behaviors are more detrimental to resilience among 29-44-year-olds (Beliefs: −0.40 in older cohort, −0.31 in younger; Behaviors: −0.59 in older cohort, −0.40 in younger).
- Having more loose connections and bridging capital benefits older cohorts more (+0.27 vs. + 0.20 for the younger group).
- Possessing close connections in discussions and friendships with different groups negatively impacts resilience more among 18-28-year-olds (−0.44 versus −0.36 among older respondents).
- Additionally, confronting hate is far more beneficial to overall resilience for 18-28-year-olds than for the older demographic (+0.74 versus +0.57 among 29–44).

Identity factors were also less important contributors to resilience among 18–28 years olds than among 29-44-year-olds (Religion and Ethnicity Identity: +0.63 for youth compared to +1.33 for the older cohort; Sexual and Gender Identity: +0.37 for youth compared to +0.58; Gamer Identity: +0.13 for youth compared to +0.55). The substantial variance in the contribution of gamer identity complexity to resilience among the different ages is particularly interesting. The general pervasiveness of games has, perhaps, made them less of a point of identity distinction among younger audiences. Or, possibly, another explanation should be examined in future research: there is insufficient data to explain the difference fully from this study.

The resilience distribution across age groups implies that while most individuals remain in the moderate resilience category, age differences may influence how gamers perceive and confront extremism. The slightly higher resilience in the 29–44 group in the “High” category could be due to increased life experience, social network diversity, and potentially more extensive exposure to different viewpoints. Conversely, the negative overall score for older gamers indicates some risk factors exogenous to this study that may be specific to that cohort.

### Gendered dynamics of resilience

Both male and female respondents predominantly fall into the “Mid” resilience group, with a slightly higher percentage of males (77.89%) than females (76.31%). Slightly more males are in the “High” resilience category (15.67%) compared to females (14.64%). However, once grouped into the “High” resilience group, women exhibit a slightly higher mean resilience score (1.59) than men (1.57), suggesting that when women exhibit strong resilience, they may be even more resistant to VE than their male counterparts.

Conversely, the mean resilience scores for the “Low” and “Mid” categories are somewhat more negative for females (−0.877 for “Low” and −0.245 for “Mid”) than for males (−0.867 for “Low” and −0.201 for “Mid”). This indicates that women in the lower resilience brackets might experience more vulnerability, which could be due to multiple gender dynamics or simply that they experience other exogenous risk factors, including far higher rates of targeted hate and harassment online, as well as gender-based violence facilitated by digital tools that undermine the presence of protective factors for males (Baekgaard, 2024).

The overall values for males (0.03) and females (−0.03) reveal an interesting contrast. While males have a slightly positive overall resilience score, females display a somewhat negative one, suggesting that the general resilience against VE might be lower among females in the gaming community. We may find some explanation for this when looking at the gendered differences among specific resilience factors, including that:

- Confronting hate contributes more to overall resilience for females (+0.66 F / +0.47 M).
- Gamer identity complexity contributes *more than three times as much* to resilience for females (+0.42 F / +0.13 M).
- Holding stronger cultural identity and connectedness factors linked to gender identity and expression, along with sexual orientation, contributes more to resilience for females than males (+0.52 F / +0.45 M).
- Religious and ethnic identity contributes less to resilience for females (+0.95 F / +0.1.177 M).
- Engaging in harassment is less impactful to overall resilience for females (−0.49 F / −0.59 M).

The gender differences in resilience highlight a nuanced reality in gaming spaces that intersect with the complex gendered inequalities that individuals bring with them into their gamer identities. Males show a marginally stronger overall resilience against VE, particularly regarding the distribution across resilience categories. However, females in the “High” resilience group exhibit slightly higher mean scores, indicating that can be quite robust when they develop resilience. The negative total score for females may reflect broader challenges they face in digital spaces, such as higher rates of harassment, gender-based violence, or exclusion from specific gaming communities.

This pattern underlines the importance of tailoring interventions and support mechanisms to address gender-specific vulnerabilities in digital environments, promoting inclusivity and strengthening resilience pathways for both males and females.

### The role of gender-based harassment in reducing resilience

The overall lower resilience among females in the “Low” and “Mid” categories potentially points to the negative impact that gender-based harassment and exclusion from gaming communities have on their ability to resist extremist narratives. Females in gaming spaces often experience higher levels of toxicity, misogyny, and exclusion compared to their male counterparts (Kelly et al., 2021; Whakaatu, 2024). This can contribute to feelings of alienation, which may lower their resilience to extremist content and violent ideologies. Additionally, the negative relationship between Bridging Capital 2 (close social ties across diverse identity groups) and resilience among female gamers suggests that, for women, engagement with other communities may not always foster resilience, potentially due to the presence of ingrained sexism or exclusionary norms in these communities.

To address these dynamics, interventions must focus on creating safer, more inclusive spaces for female gamers. Strategies such as gender-sensitive moderation protocols, the promotion of female role models in gaming, and the fostering of supportive online communities could provide protective mechanisms to strengthen resilience among female gamers. Additionally, platforms should implement anti-harassment measures tailored to address the specific vulnerabilities faced by female players. In focusing on addressing the gendered inequalities of gaming spaces to increase resilience, a result may be increased resilience for all gamers, as the allowance of hate-based harassment fundamentally harms the in- and out-groups (Wallner et al., 2023).

### Active confrontation as a resilience factor for women

Interestingly, the findings indicate that confront hate is decisive in building resilience among female gamers. This suggests that women may view taking an active stance against harassment or violence as an essential aspect of their resilience. In practice, female gamers may benefit from support systems that empower them to speak out against harmful behaviors or extremist content in their gaming communities. This could include tools for reporting harassment, opportunities for participating in collective action against toxic behavior, or initiatives that encourage women to take on leadership roles within gaming spaces.

## Conclusion - implications for building resilience against violent extremism in gaming spaces

The study highlights several factors that contribute to resilience against VE in gaming spaces, offering valuable insights for policymakers, game developers, and preventing and countering violent extremism (P/CVE) practitioners.

### Promoting inclusive community-building to Foster resilience

The role of Bridging Capital (loose social ties with diverse identity groups) in fostering resilience is particularly significant. Gamers who have positive interactions with players from different backgrounds—whether ethnic, religious, or otherwise—demonstrate higher levels of resilience to extremist narratives. This suggests that creating inclusive gaming environments where players from diverse backgrounds can engage meaningfully is crucial for building digital resilience. Game developers, policymakers, and platforms should prioritize cross-cultural and cross-identity engagement, as well as facilitating community norm building that allows for inclusive gaming communities, to counter extremist ideologies in gaming spaces.

However, the data also highlights that close social ties (Bridging Capital 2) can sometimes harm resilience, particularly for female gamers and in specific cultural contexts such as Indonesia. This suggests that “echo chambers” within gaming spaces, where players are insulated from diverse perspectives, may reinforce exclusionary or extremist attitudes. Therefore, efforts to foster resilience should also encourage open, inclusive dialogue rather than creating insular, homogeneous groups. Safe spaces matter – but should not be the exclusive option provided to users.

### The role of cultural identity in strengthening resilience

Cultural identity also emerges as an essential factor in resilience. Players who have a strong sense of their own cultural and religious identities tend to demonstrate higher resilience to extremist narratives. This finding underscores the importance of allowing gamers to express their cultural identities freely within gaming spaces. In particular, players from countries like the United States and Australia, where ethnic and religious identity played a significant role in resilience scores, could benefit from initiatives that celebrate diversity and promote pride in cultural heritage.

P/CVE practitioners could work with game developers to create in-game content that reflects diverse cultural experiences and fosters pride in one’s identity. By doing so, gaming platforms can serve as spaces where players feel empowered in their cultural identities, rather than alienated, thus increasing their resilience to extremist ideologies that seek to exploit feelings of marginalization.

### Empowering gamers to confront extremism

Finally, the role of active confrontation in resilience is noteworthy across gender and age groups. Gamers who actively confront harmful behaviors or extremist content in their communities have higher resilience scores. This finding suggests that empowering players to take a stand against extremism—through reporting tools, community moderator support systems, or peer-led initiatives—can significantly bolster their resilience. Platforms should invest in tools that make it easier for players to report harmful content and offer incentives for players who actively contribute to creating a safer, more inclusive gaming environment.

## Data Availability

The raw data supporting the conclusions of this article will be made available by the authors, without undue reservation.
