# Supplementary material for: Building resilience against violent extremism digitally: trialing a new gender-based approach among gamers
Source: Front Psychol. 2025 Jul 28;16:1537492. doi: 10.3389/fpsyg.2025.1537492 (PMC12337677; doi:10.3389/fpsyg.2025.1537492)
Supplement: Supplementary file 1 [file Data_Sheet_1.docx]

Building Resilience Against Violent Extremism Digitally: Trialing a New Gender-Based Approach Among Gamers

**Supplementary Material**

**Authors**: Galen Lamphere-Englund, Mike Wilson, Dr. Jessica White, Claudia Wallner, Dr. Rachel Kowert, Nitchakarn Kaewbuadee, Petra Regeni, Alex Newhouse

### Annex One: Survey Instrument

This annex provides the survey instrument used for this project.

# **Survey Sections**

## **Informed Consent**

Hi! This survey is being administered by an independent social research agency, Love Frankie. We would like your help with a research study about online video games, especially on social challenges and harms being faced by players and their resilience towards these challenges. This piece of research is important in order for us to understand the diverse needs and challenges faced by people playing online games like yourself.

This study involves completing an anonymous survey which should take approximately 10 minutes of your time. If you are happy to complete this survey, ticking the box below will show your consent and start the survey. As this survey is anonymous, the research team will not know whether you have participated or what answers you provided.

If you decide to take part in this survey, you will not receive any direct benefits; however, your participation will contribute to knowledge in this area of research.

It is expected that taking part in this study will not cause you any psychological discomfort and/or distress, however, should you feel uncomfortable you can leave the survey at any time or contact the following resources for support:

All information collected for this study will be stored securely on GSuite UAC permissions Google Drive folders. Survey data is hosted on AWS-hosted UAC controlled Pollfish database with raw data being transferred to Google Drive folders through an encrypted link. The information collected will be analysed and written up as part of a research consortium led by the Royal United Services Institute.

If you have any questions or concerns, you can contact research@lovefrankie.co.

1. Y/N

## **Demographics and gameplay screener**

## ***Demographics***

**Gender (Leave out for Indonesia)**

| Male |
| --- |
| Female |
| Transgender |
| Intersex |
| Non-Binary |
| Choose not to identify |

**Sexual orientation** **(Leave out for Indonesia)**

| Straight |
| --- |
| Lesbian |
| Gay |
| Bisexual |
| Queer |
| Choose not to identify |

## ***Gameplay***

S1. Do you play online multiplayer video games? These can include games on PC, XBox, Playstation, Nintendo and other consoles, as well as mobile phones if you play with other players online. If you *only* play single-player games or *only* play with people who you know offline, then please select No.

| Yes |  |
| --- | --- |
| No | **> Screen out** |

## ***Games and media consumption***

1. What types of games do you primarily play online? **(Select all that apply to you)**

| Sandbox games |
| --- |
| Shooters (FPS and/or TPS) |
| Multiplayer Online Battle Areana (MOBA) |
| Role Playing Games (RPG, APRG, others) |
| Real Time Strategy (RTS) or Turn Based Strategy games |
| Survival or horror |
| Sports |
| Other, please specify |

1. How many hours a week do you play online video games?

| 0-3 hours |
| --- |
| 3-8 hours |
| 8-20 hours |
| 20-40 hours |
| 40+ hours |

1. Which, if any, of these apps or sites do you use to send messages or communicate? **(Select all that apply to you)**

| Steam (Messenger) |
| --- |
| Twitch |
| Discord |
| (Facebook) Messenger |
| Instagram (Direct Message) |
| Signal |
| Telegram |
| DLive |
| Odysee |
| Twitter |
| Threads |
| Other, please specify |

## ***Digital Resilience***

## **Identity**

1. For each of the below questions, please select how much or how little the statement applies to you.

| Statement | Agree | Somewhat Agree | Somewhat Disagree | Disagree |
| --- | --- | --- | --- | --- |
| My gamer identity is important to me |  |  |  |  |
| Online gaming or participation in gaming communities are important to me |  |  |  |  |
| I believe the globalisation of gaming spaces makes my communities worse off |  |  |  |  |
| The way I am living my life is guided by the traditions, beliefs, habits, and norms of my culture/ethnic group |  |  |  |  |
| Religion is important for me and guides me on how to behave in society |  |  |  |  |

1. My gamer identity represents the following... **(Select all that apply to you)**:

| Age |
| --- |
| Class |
| Ethnicity/race |
| Nationality |
| Religion |
| Gender identity/expression |
| Sexual orientation |

1. I feel comfortable playing with people of different identities, including different … **(Select all that apply to you)**:

| Ages |
| --- |
| Class |
| Ethnicity/race |
| Nationality |
| Religion |
| Gender identity/expressions |
| Sexual orientation |
| All of the above |
| I prefer to play with people that share my identity (EXCLUSIVE) |

1. I have actively sought/found communities based on similarity to my... **(Select all that apply to you)**:

| Age |
| --- |
| Class |
| Ethnicity/race |
| Nationality |
| Religion |
| Gender identity/expression |
| Sexual orientation |

## **Bridging Capital / Outgroup contact / Tolerance**

1. For these next questions, please imagine that you’re online with people from various backgrounds and beliefs. Select how much you agree or disagree with the following statements:

| Statement | Agree | Somewhat Agree | Somewhat Disagree | Disagree |
| --- | --- | --- | --- | --- |
| It’s not easy for me to trust people online who are different than me (for example, other genders, ethnicities, cultures, or religions) |  |  |  |  |
| I regularly engage in conversations online with people with different identities or backgrounds |  |  |  |  |
| I have strong and meaningful friendships online with friends from different identities than mine |  |  |  |  |

## **Exposure to Online Harms**

**Now, we would like to ask if you have experienced any of these behaviours in games or in gaming-related spaces online (such as forums, chatrooms, Discord servers, livestreams, and so on).**

1. I **have witnessed** people being harassed based **on their**... **(Select all that apply)**:

| Religion |
| --- |
| Ethnicity/race |
| Gender Identity |
| Sexual orientation |
| Age |
| Class |
| Nationality |
| I have not witnessed any harassment |

1. I **have been harassed** based **on my**... **(Select all that apply)**:

| Religion |
| --- |
| Ethnicity/race |
| Gender Identity |
| Sexual orientation |
| Age |
| Class |
| Nationality |
| I have not experienced any harassment |

1. I **have bullied** or harassed people based **on their**... **(Select all that apply)**:

| Religion |
| --- |
| Ethnicity/race |
| Gender Identity |
| Sexual orientation |
| Age |
| Class |
| Nationality |
| I have not bullied |

1. I feel unable to join some communities based on parts of my identity.

| Yes |  |
| --- | --- |
| No | **> Skip to Q14** |

1. If so, which part of your identity?

| Religion |
| --- |
| Ethnicity/race |
| Gender Identity |
| Sexual Orientation |
| Age |
| Class |
| Nationality |
| Other (please specify) |

1. Select how much you agree or disagree with the following statements:

| Statement | Agree | Somewhat agree | Agree | Somewhat agree |
| --- | --- | --- | --- | --- |
| I feel that harassment based on identity is commonplace in online gaming and adjacent spaces |  |  |  |  |
| I can tell the difference between commonplace toxicity and extremist content online |  |  |  |  |
| I can tell the difference between online sexism and misogyny |  |  |  |  |

1. Select the level of social acceptance you perceived from the following statements:

| Statement | Unacceptable | Normalized | Culturally Acceptable | Justified |
| --- | --- | --- | --- | --- |
| I think **toxicity** in the gaming space is: |  |  |  |  |
| I think **extremism** in the gaming space is: |  |  |  |  |

1. Have you seen any of the following extremist content in gaming spaces **(Select all that apply)**:

| Images, videos or symbols that promote extremism |
| --- |
| People endorsing violence against a particular group |
| Suggesting that people join a group based on extremist ideas |
| Suggesting that people donate to support a group or individual with extremist ideas |

1. Where have you found the most extreme harassment in gaming or gaming-adjacent spaces? Be as specific as possible (which games or platforms):

**(Open-end)**

## **Ability to respond and report / Linking Capital**

1. Which of the following best describes you when it comes to reporting harmful content?

| Statement | In Computer Games | In Console Games | On Gaming-adjacent platforms | On all of the above | On none of the above |
| --- | --- | --- | --- | --- | --- |
| I know how to report harmful content online… |  |  |  |  |  |
| I have reported harmful content in the last year… |  |  |  |  |  |

1. Select how much you agree or disagree with the following statements:

| Statement | Agree | Somewhat Agree | Somewhat Disagree | Disagree |
| --- | --- | --- | --- | --- |
| I feel my voice is heard when reporting harmful content |  |  |  |  |

## Allyship

1. When was the last time you have stood up for someone else being harassed online?

| Statement | In the last week | In the last month | In the last year | Never |
| --- | --- | --- | --- | --- |
| I have stood up for someone else being harassed online from my own *community* |  |  |  |  |
| I have stood up for someone else being harassed online from a *different community* |  |  |  |  |
